# Organogenesis of Ileal Peyer's Patches Is Initiated Prenatally and Accelerated Postnatally With Comprehensive Proliferation of B Cells in Pigs

**DOI:** 10.3389/fimmu.2020.604674

**Published:** 2020-12-04

**Authors:** Mutsumi Furukawa, Shun Ito, Shunichi Suzuki, Daiichiro Fuchimoto, Akira Onishi, Kanae Niimi, Katsuki Usami, Guoyao Wu, Fuller W. Bazer, Kouetsu Ogasawara, Kouichi Watanabe, Hisashi Aso, Tomonori Nochi

**Affiliations:** ^1^ International Education and Research Center for Food and Agricultural Immunology, Graduate School of Agricultural Science, Tohoku University, Sendai, Japan; ^2^ Division of Animal Science, Institute of Agrobiological Sciences, National Agriculture and Food Research Organization, Tsukuba, Japan; ^3^ Department of Animal Science and Resources, Nihon University College of Bioresource Sciences, Fujisawa, Japan; ^4^ Department of Animal Science, Texas A&M University, College Station, TX, United States; ^5^ Department of Immunobiology, Tohoku University Institute of Development, Aging and Cancer, Sendai, Japan; ^6^ International Research and Development Center for Mucosal Vaccines, The Institute of Medical Science, The University of Tokyo, Tokyo, Japan

**Keywords:** Peyer's patches, ileum, organogenesis, B cells, pigs

## Abstract

Morphogenesis and differentiation of organs is required for subsequent functional maturation. The morphological features of Peyer's patches vary among species. In pigs, they develop extensively in the ileum as ileal Peyer's patches (IPPs). However, the role of IPPs in the porcine immune system remains to be elucidated because of a lack of complete understanding of IPP organogenesis. Results of the present study revealed that development of porcine IPPs is initiated prenatally between embryonic days 76 and 91. The process of IPP organogenesis is concomitant with increased transcriptional patterns of CXCL13 and CCL19. IPPs undergo further development postnatally by forming central, marginal, and subepithelial zones. Importantly, a large number of proliferating B cells and apoptotic cells are found in porcine IPPs postnatally, but not prenatally. The expression level of IgM in proliferating B cells depends on the zone in which distinct B cells are separately localized after birth. Specifically, IgM^+^ cells are predominantly found in the central zone, whereas IgM^-/low^ cells are abundant in the marginal zone. Importantly, the cellular feature of IPPs differs from that of mesenteric lymph nodes (MLNs) where such distinct zones are not formed both prenatally and postnatally. Our findings suggest that IPPs (not MLNs) in postnatal pigs are involved in complementing functions of the primary lymphoid tissue that promotes the differentiation and maturation of B cells.

## Introduction

Numerous studies using murine embryos and neonates to address the molecular and cellular mechanisms of lymphoid tissue development have concluded that the organogenesis of most lymphoid tissues is initiated prenatally during the embryonic stages of development (1–6). Postnatal stimulation of lymphoid tissues by foreign antigens, such as those on microorganisms, further accelerates their structural formation and functional maturation (7, 8). Peyer's patches (PPs), which are gut-associated lymphoid tissues, play a pivotal role in inducing immune responses in the gastrointestinal tract. During fetal development, T and B cells migrate into the anlagen where PPs develop in response to several chemokines (i.e., CXCL13, CCL19, and CCL21) and adhesion molecules (i.e., VCAM-1 and ICAM-1), both of which are expressed in the PP anlagen (9–11). According to results of previous studies focusing on the intestinal immune system in mice and humans, PPs have been considered secondary lymphoid tissue in the gastrointestinal tract (12, 13). Foreign antigens, including intestinal microorganisms, are taken into PPs through antigen-sampling microfold cells (M cells) and processed by dendritic cells and macrophages for the presentation of antigen-derived peptides in association with MHC class II. T cells bearing antigen-specific T cell receptors are activated by such antigen-presenting events and stimulate B cells in an antigen-specific manner to initiate immune responses, including antibody production (14, 15). However, it is important to note that the definition of PPs depends on the species as they are considered to be primary lymphoid tissues in the ileum of sheep and cattle (16–19). Pigs, which are important industrial animals, have been utilized as a suitable animal model for humans because of metabolic, physiological and anatomical similarities between the two species (20). However, the role of porcine PPs in immune function remains yet to be elucidated.

Comparative analyses of the organogenesis of PPs among multiple species have demonstrated that several species of animals (e.g., pigs, cattle, sheep, and horses) develop two types of PPs (21). The differences between these distinct PPs are size, shape, and location of development in the small intestine. Those that develop in the terminal ileum are referred to as ileal Peyer's patches (IPPs). IPPs contain dense follicles that extend up to 2 m. Others are scattered in the jejunum and are referred to as jejunal Peyer's patches (JPPs). JPPs form a patch structure containing several lymphoid follicles (22). The frequency of B cells in IPPs is much greater than that in JPPs, since IPPs develop large follicular regions and few interfollicular regions containing abundant B cells and a few T cells, respectively (23). In pigs, the organogenesis of JPPs and IPPs may be independently regulated since follicular regions of JPPs further develop postnatally upon colonization by intestinal microorganisms, whereas those of IPPs remain the same, even in pigs bred in a germ-free environment (24). However, the fundamental differences in immune functions between JPPs and IPPs are poorly understood. This may be due to an oversight in a broader field of immunology that focuses on mice and humans having a single type of PPs.

It was revealed by *in vivo* research that the surgical resection of IPPs in piglets did not influence subsequent immune development. In fact, the number of antibody-containing cells in the spleen and peripheral lymph nodes, and the concentration of immunoglobulins in serum and mucosal secretions are not altered in the absence of IPPs (25). It is suspected that IPPs in pigs, unlike those in sheep and cattle, do not act as a primary lymphoid tissue from which lymphocytes, including B cells, originate (26). Additionally, further studies demonstrated that IPPs are involved in producing naturally derived T cell-independent IgA, especially early in life (27). IPPs in pigs appear to be the site for initial immune reactions that produce undiversified IgA in the absence of T cell help. When JPPs develop postnatally, they initiate immune responses for producing diversified IgA with support from T cells. In porcine IPPs, CD21^+^ B cells are classified as IgM^+^ and IgM^−^ based on flow cytometry analysis, suggesting that IgM^−^ cells undergo immunoglobulin class switching from IgM to other classes, such as IgA. IgM^−^ cells are predominantly found in large numbers in the marginal zone of follicular regions in IPPs (23). However, it must be emphasized that few class-switched IgA^+^ cells are present in the follicular and subepithelial dome (SED) regions and in the follicle-associated epithelium (FAE) (28), indicating that IgM^−^ cells are not histologically identical to IgA^+^ cells. Therefore, elucidation of the immunological status of each immune cell subset in IPPs is justified to gain deeper insight into the importance of IPPs in the porcine immune system.

Results of our present research have demonstrated three main findings in pigs: 1) initiation of IPP organogenesis between embryonic days 76 and 91 concomitant with increased expression of CXCL13 and CCL19; 2) acceleration of postnatal IPP development while increasing the number of Ki-67^+^ proliferating cells and TUNEL^+^ apoptotic cells in the follicular regions and MHC class II^+^ antigen-presenting cells in the SED regions; and 3) local expansion of IgM^−/low^ cells found postnatally in the marginal zone of follicular regions without undergoing immunoglobulin class switching. These results suggest that IPPs in pigs possess unique features that are not found in other species and function to adapt to pig-specific changes in the intestinal environment early in life.

## Materials and Methods

### Animals and Samples

Pregnant pigs were sacrificed on fetal days 76 (n = 5), 91 (n = 5), and 110 (n = 5) to provide fetuses and piglets were sacrificed on postnatal day 9 (n = 4) to harvest duodenum, ileum, and mesenteric lymph nodes. Embryonic ages were based on the known dates when pigs were insementated artificially. The sampling dates from fetuses and piglets were determined based on the findings obtained from a past study using mice and a consideration of the difference in gestation period between mice and pigs (6). All experiments were conducted according to protocols approved by the institutional animal care and use committee of the Institute of Agrobiological Sciences, National Agriculture and Food Research Organization (NARO).

### Histological Analyses

Tissues harvested from fetuses and neonates were fixed in 4% (w/v) paraformaldehyde (Nacalai Tesque) overnight at 4°C and embedded in paraffin. To analyze the development of lymphoid structures, tissue sections (5 μm) were dewaxed and stained with hematoxylin and eosin (H&E). Some tissue sections of the ileum and mesenteric lymph nodes were subjected to immunohistochemistry to detect the presence of B and T, antigen-presenting, and proliferating cells. Specifically, the sections were treated with REAL Target Retrieval Solution (DAKO) for 40 min at 98°C or 0.05% (w/v) of proteinase (Sigma) for 3 min at 37°C (Sigma) to retrieve antigen and incubated with 0.5% (w/v) blocking reagent (PerkinElmer) for 30 min at room temperature (RT) to block non-specific binding of antibodies. The sections were then treated with primary antibodies overnight at 4°C followed by secondary antibodies for 1 h at RT. Primary antibodies used in this study were rabbit anti-CD20 (1:100, polyclonal, Biocare Medical), rabbit anti-CD3 (1:100, SP7, Abcam), goat anti-IgM (100 ng/ml, polyclonal, Bethyl Laboratories), mouse anti-MHC class II DQ (1:100, K274.3G8, Abcam), and rabbit anti-Ki-67 (1:200, polyclonal, Abcam), all of which are commercially available for detecting target antigens in pigs. Isotype control antibodies used to confirm the specificity of primary antibodies were universal negative control serum (undiluted, Biocare Medical), rabbit IgG isotype control (1:100, EPR25A, Abcam), mouse IgG1 isotype control (1:100, MOPC-21, Abcam), and goat IgG isotype control (100 ng/ml, polyclonal, Bethyl Laboratories). Secondary antibodies used in this study were Histofine Simple Stain MAX PO (R) and (G) (undiluted, Nichirei Biosciences), horse radish peroxidase (HRP)-conjugated donkey anti-goat IgG (1:2,000, Jackson ImmunoResearch), Alexa Fluor 647-conjugated donkey anti-rabbit IgG (1:200, Jackson ImmunoResearch), and Alexa Fluor 647-conjugated donkey anti-mouse IgG (1:200, Jackson immune Research). The enzyme activity of HRP was visualized using either 3,3′-diaminobenzidine tetrahydrochloride (Dojindo Molecular Technologies) or the TSA Plus fluorescein system (PerkinElmer). The sections were finally counterstained with either hematoxylin or DAPI. Apoptotic cells were detected using the DeadEnd Colorimetric TUNEL System (Promega) according to the manufacturer's instructions. Tissue images were obtained using either a BX63 (Olympus), BZ-9000 (Keyence), or BZ-X800 (Keyence) microscope.

### Quantitative RT-PCR

Total RNA was extracted from duodenal and ileal tissues using the ReliaPrep RNA Tissue Miniprep System (Promega). PrimeScript RT reagent Kit (Perfect Real Time) (Takara) was used to synthesize cDNA from total RNA. Real-time PCR was conducted using TB Green Premix Ex Taq II (Takara) to quantify the expression of mRNAs coding for *cxcl13*, *ccl19*, *ccl21*, and *gapdh*. Glyceraldehyde-3-phosphate dehydrogenase was used as an endogenous control to normalize expression levels. All primers were designed using the Takara perfect real-time support system.

### Statistical Analysis

**Figure 1:**
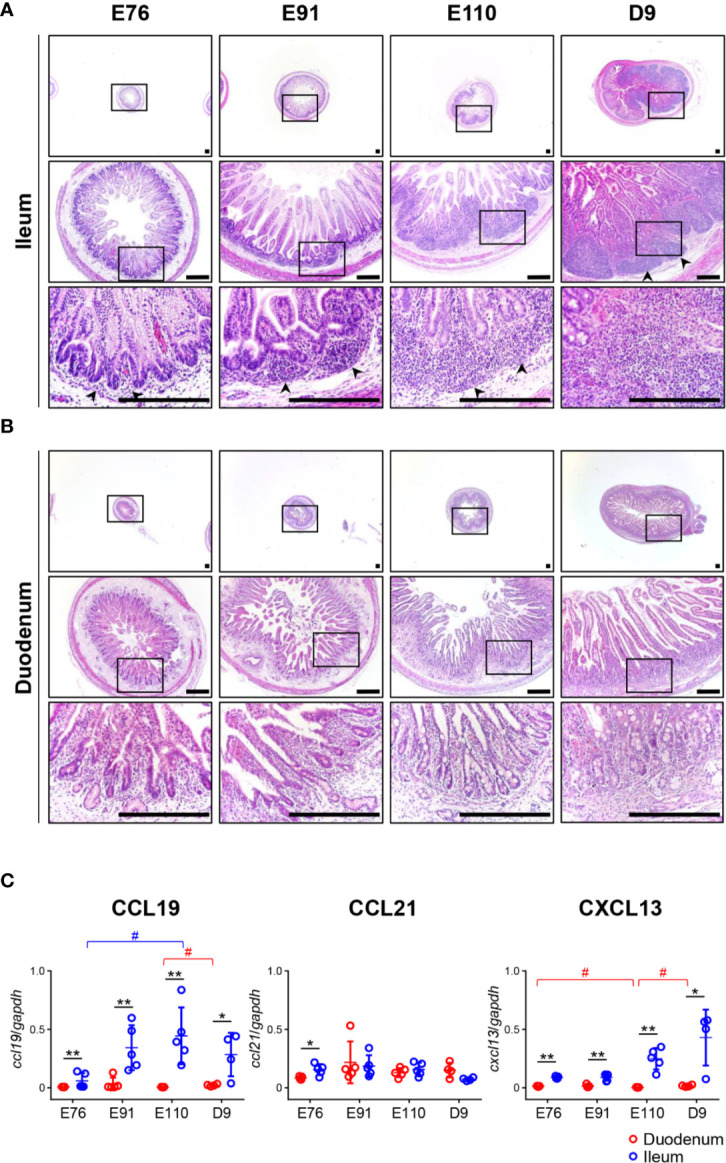
Organogenesis of Peyer’s patches (PPs) was initiated prenatally in the ileum and not the duodenum. **(A)** In the ileum, the formation of lymphoid aggregates was initiated between E76 and E91, and their size increased between the fetal and neonatal stages. **(B)** In the duodenum, there was no accumulation of infiltrated cells throughout the experiment. **(C)** Quantitative RT-PCR analyses demonstrated that the expression of *ccl19* and *cxcl13* mRNAs in the ileum were consistently higher than those in the duodenum. Tissue sections shown in **(A, B)** were stained by H&E. Scale bars = 250 μm. *^(or #)^ *p *< 0.05, **p < 0.01. Arrowheads: follicular regions (or PP anlagen).

**Figure 2:**
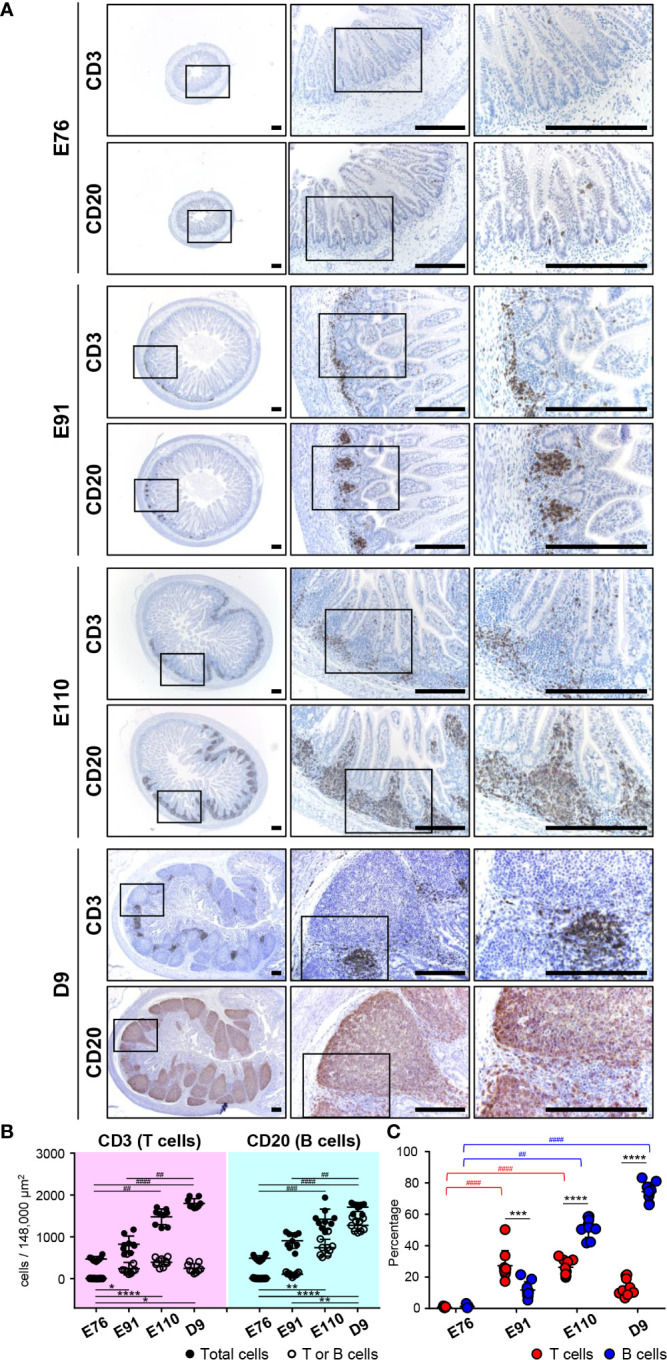
B and T cells accumulated gradually in follicular and interfollicular regions of IPPs throughout organogenesis, respectively. **(A)** CD3^+^ cells (T cells) and CD20^+^ cells (B cells) were extremely rare at E76, whereas they were abundant in the ileum at E91. B cells thereafter formed the follicular region by further increasing the number in IPPs between E91 and D9, whereas T cells formed the interfollicular region without a dramatic increase in number. **(B)** The numbers of T, B, and total cells not including cells in the epithelial layer were demonstrated. Blood vessels and muscle layers were counted in the three defined areas from three distinct tissue sections. Multiple comparison tests were conducted on the number of total cells and T or B cells. **(C)** The frequency of CD3^+^ T cells or CD20^+^ B cells, among all cells, was determined to compare the abundance of T and B cells in IPPs. Multiple comparison tests were conducted on the percentages of T or B cells throughout the experiment. *t*-tests were conducted to compare the difference between the percentages of T and B cells in each stage of fetal and neonatal development. Tissue sections shown in **(A)** were stained by immunohistochemistry using anti-CD3 and anti-CD20 antibodies. Scale bars = 250 μm. *p < 0.05, **^(or ##)^ *p *< 0.01, ***^(or ###)^ *p *< 0.001, ****^(or ####)^ *p *< 0.0001.

**Figure 3:**
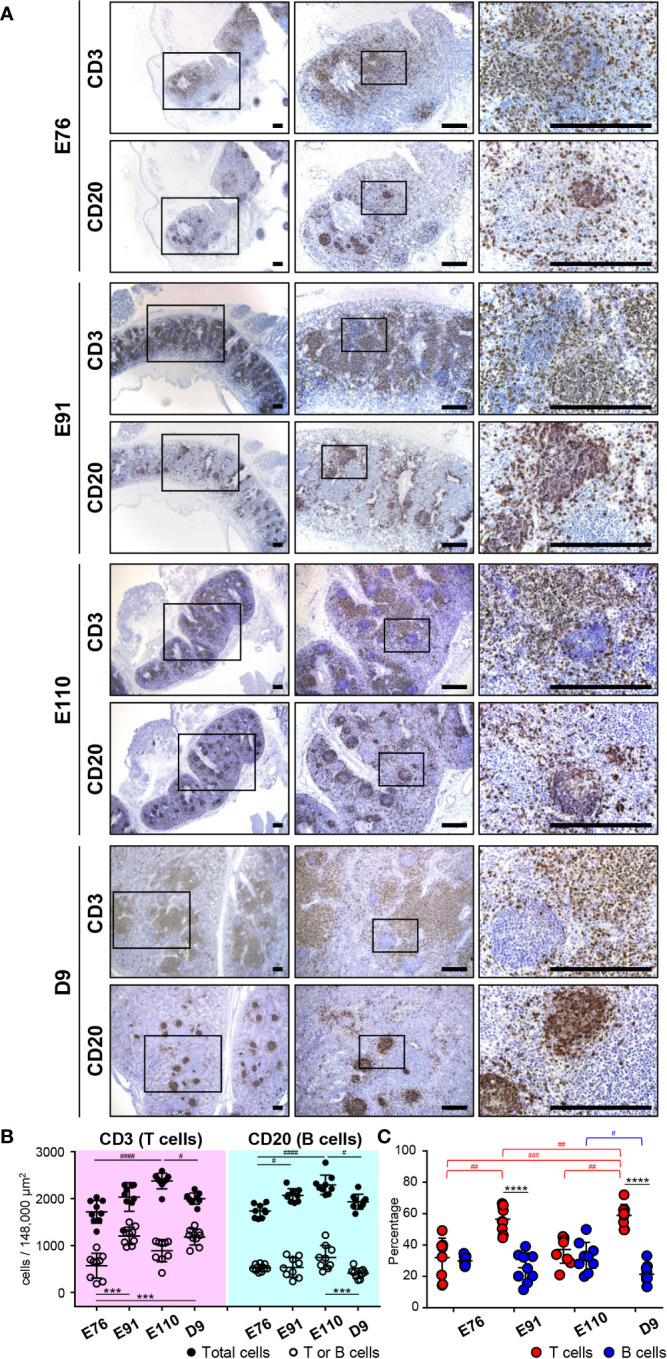
Organogenesis of MLNs was initiated earlier than that for IPPs. **(A)** Immunohistochemical analysis using anti-CD3 and anti-CD20 antibodies revealed that CD3^+^ cells (T cells) and CD20^+^ cells (B cells) were abundant in MLNs throughout the experiment from E76 to D9. Specific localization of T and B cells formed around E110 since most B cells were present in the cortical nodules, and T cells were found predominantly in the cortex at E110 and D9, but not at E76 and E91. **(B)** The numbers of T, B, and total cells were counted in the three defined areas from three distinct tissue sections. Multiple comparison tests were conducted on the number of total cells and T or B cells. **(C)** The frequency of CD3^+^ T cells and CD20^+^ B cells, among all cells, was determined to compare the abundance of T and B cells in MLNs. Multiple comparison tests were conducted on the percentages of T and B cells throughout the experiment. *t*-tests were conducted to compare the differences between the percentages of T and B cells in each stage. Tissue sections shown in **(A)** were stained by immunohistochemistry using anti-CD3 and anti-CD20 antibodies. Scale bars = 250 μm. ^#^ *p *< 0.05, ^##^ *p* < 0.01, ***^(or ###)^ *p *< 0.001, ****^(or ####)^ *p *< 0.0001.

Statistical analyses were conducted by using the *t*-test for results in **Figures 1C**, **2C**, **3C**, and **6C** and by one-way ANOVA with the Kruskal–Wallis test for results in **Figures 1C**, **2B**, **2C**, **3B**, and **3C** using Prism 7 software (GraphPad).

## Results

### Organogenesis of IPPs in Pigs Was Initiated Prenatally and Accelerated Postnatally

Tissue sections prepared from the duodenum and ileum were first analyzed by H&E staining. No obvious lymphoid aggregates were observed in the ileum at E76 although a few cells had infiltrated into the tissue (**Figure 1A**). The recruitment of infiltrated cells between the epithelial layer and muscularis mucosa was accelerated between E76 and E91 to form lymphoid aggregates (**Figure 1A**). The size of lymphoid aggregates in the ileum increased during fetal development due to subsequent accumulation of infiltrated cells into the anlagen of IPPs, and such structures underwent further development after birth (**Figure 1A**). In the duodenum, no lymphoid aggregates were detected throughout the experimental period (**Figure 1B**). To understand the molecular mechanism by which IPPs developed, the expression levels of genes encoding CXCL13 and CCL19/CCL21 were determined as they promote B and T cell migration, respectively, toward the anlagen of lymphoid tissues, including IPPs. When mRNAs extracted from the duodenum and ileum at E76, E91, E110, and D9 were subjected to quantitative RT-PCR analyses, the expression levels of CXCL13 and CCL19 in the ileum were confirmed to be consistently higher than those in the duodenum throughout the experimental period (**Figure 1C**). On the contrary, the expression of CCL21 mRNA in the ileum at E76 was slightly, but significantly higher than that in the duodenum; however, there was no difference in expression between the duodenum and ileum at E91, E110, and D9 (**Figure 1C**). These results suggested that CXCL13 and CCL19 play a key role in initiating the development of IPPs by recruiting B and T cells into the anlagen.

### Follicular (B-cell) and Interfollicular (T-cell) Regions Were Formed in IPPs by an Unrelated Increase in B and T Cells

To investigate the formation of follicular and interfollicular regions of IPPs that mostly comprise B and T cells, respectively, ileal tissues harvested at E76, E91, E110, and D9 were further subjected to immunohistochemical analyses using anti-CD20 and anti-CD3 antibodies capable of detecting porcine B and T cells, respectively, in tissue sections. Consistent with the H&E-stained tissue images shown in **Figures 1A, B**, only a few CD20^+^ B cells and CD3^+^ T cells were detected at E76 in tissues that did not develop lymphoid aggregates (**Figure 2A**). By contrast, CD20^+^ B and CD3^+^ T cells were obviously found at E91 (**Figure 2A**), indicating that both cell lineages initiate migration into the anlagen of IPPs between E76 and E91. The structure of follicular and interfollicular regions was not clearly delineated at E91; however, the structures were much better organized at E110 because CD20^+^ B and CD3^+^ T cells accumulated in the anlagen of IPPs between E91 and E110 to develop follicular and interfollicular regions, respectively (**Figure 2A**). The distribution of B and T cells at E110 was comparable to that at D9, although the numbers of both cell lineages in IPPs increased dramatically between E110 and D9, especially the numbers of B cells (**Figure 2A**). Subsequent quantitative analysis demonstrated that the total number of cells in the defined areas of the ileum increased dramatically during fetal and neonatal development (**Figure 2B**). A further comparative analysis of total, CD3^+^ T, and CD20^+^ B cells indicated that the frequency of CD3^+^ T cells (27.25% ± 9.63%) was greater than that for B cells (11.79% ± 5.25%) at E91 (**Figure 2C**). However, the abundance of B cells increased significantly between E91 and D9 (51.56% ± 6.20% at E110 and 74.42% ± 5.84% at D9), whereas the abundance of T cells was not affected (**Figure 2C**). These results indicated that the development of IPPs depended on the acceleration of development of the follicular region with increasing numbers of B cells, especially after birth.

### Organogenesis of IPPs Was Distinct to That of MLNs

The developmental process for IPP was compared with that of mesenteric lymph nodes (MLNs), which drain the lymph nodes of the gastrointestinal tract. MLNs harvested at E76, E91, E110, and D9 from the same animals used for IPP analysis were subjected to immunohistochemistry using anti-CD20 and anti-CD3 antibodies to detect the presence of B and T cells. MLNs grew in size during fetal and neonatal development from E76 to D9. However, sufficient numbers of B and T cells were already present at E76 in the defined areas of MLNs (**Figure 3A**). Multiple comparison analysis demonstrated that both the numbers and frequencies of B and T cells varied slightly but significantly at time points between E76 and D9 (**Figures 3B, C**). The distribution of B and T cell was well organized in the later stages of fetal development. Specifically, B cells were mostly present in the cortical nodules, whereas T cells were predominantly found in the cortex at E110 and D9 (**Figure 3A**). By contrast, both cell lineages were more dispersed within the tissues at E76 and D91; therefore, defined cortical nodules were not present in MLNs during the early stages of fetal development (**Figure 3A**). Notably, B cells were predominant in IPPs, whereas T cells were the major population in MLNs, especially after birth (see **Figures 2C**, **3C**). These results indicated that organogenesis of IPPs and MLNs were regulated differently even though both tissues play an important role in the gastrointestinal tract.

### B Cells Proliferated Postnatally in the Follicular Regions of IPPs

**Figure 4:**
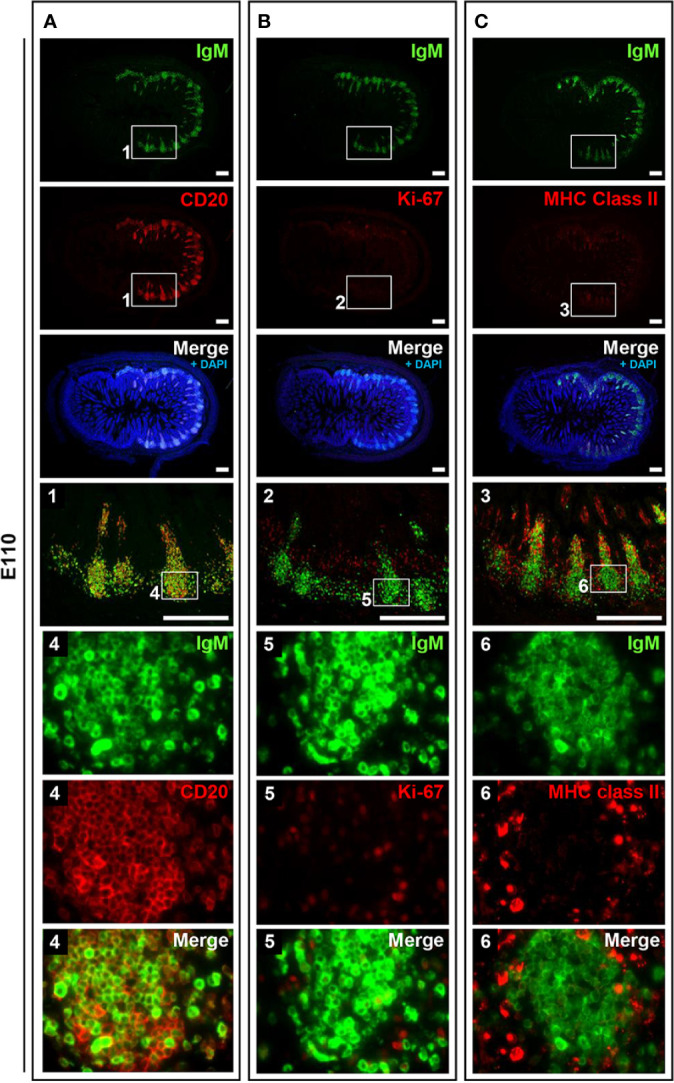
B cells did not frequently proliferate in prenatal IPPs. **(A)** CD20^+^ cells that expressed different levels of IgM were found in IPPs. **(B)** Most B cells in the follicles of IPPs did not express Ki-67. **(C)** The expression of MHC class II was sparsely detected in IPPs. Tissue sections were stained by immunohistochemistry using anti-IgM and either anti-CD20 **(A)**, anti-Ki-67 **(B)**, or anti-MHC class II **(C)**. Scale bars = 250 μm.

**Figure 5:**
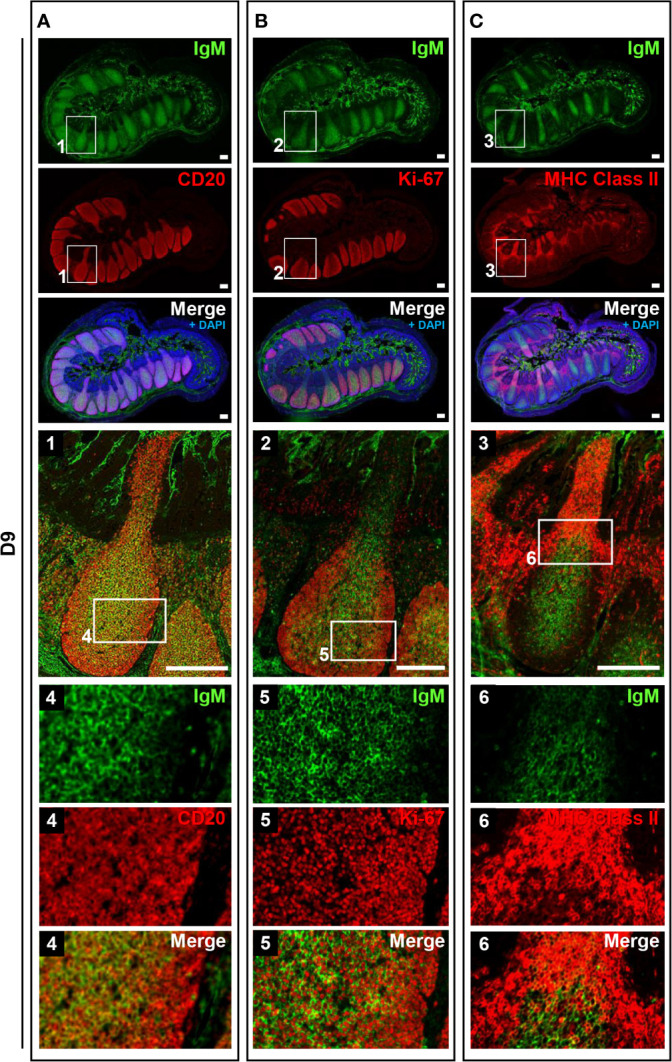
IPPs developed postnatally in the follicular region composed of high proliferating B cells. **(A)** CD20^+^IgM^+^ cells and CD20^+^IgM^−/low^ cells were distinctly observed in the central and marginal zones in the follicular regions of IPPs, respectively. **(B)** Most cells present in the central and marginal zones highly expressed Ki-67. **(C)** The expression of MHC class II was detected in the subepithelial dome region of IPPs. Tissue sections were stained by immunohistochemistry using anti-IgM and either anti-CD20 **(A)**, anti-Ki-67 **(B)**, or anti-MHC class II **(C)**. Scale bars = 250 μm.

To address the postnatal development of IPPs at the cellular level, ileal tissues sampled at E110 and D9 were further compared using immunofluorescence analyses to detect IgM and either CD20, Ki-67, or MHC class II. Consistent with our initial immunohistochemical analysis shown in **Figure 2A**, CD20 was detected identically in the follicular regions of IPPs at E110 and D9 (**Figures 4** and **5**). The expression of IgM was almost identical to that of CD20 when analyzed at a low magnification. However, high magnification images showed that the level of IgM expression in CD20^+^ cells was heterogenous in the follicular regions of IPPs at E110 (**Figure 4A**). More importantly, CD20^+^ cells were clearly classified into two types, which were distinctly localized in the follicular regions of IPPs, based on the expression level of IgM after birth. Specifically, CD20^+^IgM^−/low^ were predominantly found in the marginal zone, whereas CD20^+^IgM^+^ cells were mostly found in the central zone of IPPs at D9 (**Figure 5A**). CD20^+^ B cells were highly proliferative at D9 as evidenced by the expression of Ki-67, a cellular marker for proliferating cells (**Figure 5B**). By contrast, little or no expression of Ki-67 was detected in CD20^+^ B cells at E110 regardless of the heterogenous IgM expression (**Figure 4B**). Importantly, cells abundantly expressing MHC class II were predominantly found in the SED region under the follicular-associated epithelium at D9, but not at E110 (**Figures 4C** and **5C**). These results indicated that the structure of IPPs underwent further development postnatally due to the local expansion of B cells in the follicular regions and the increased infiltration of antigen-presenting cells in the SED region.

### MLNs Possessed Phenotypic Characteristics of Secondary Lymphoid Tissue Unlike IPPs

**Figure 6:**
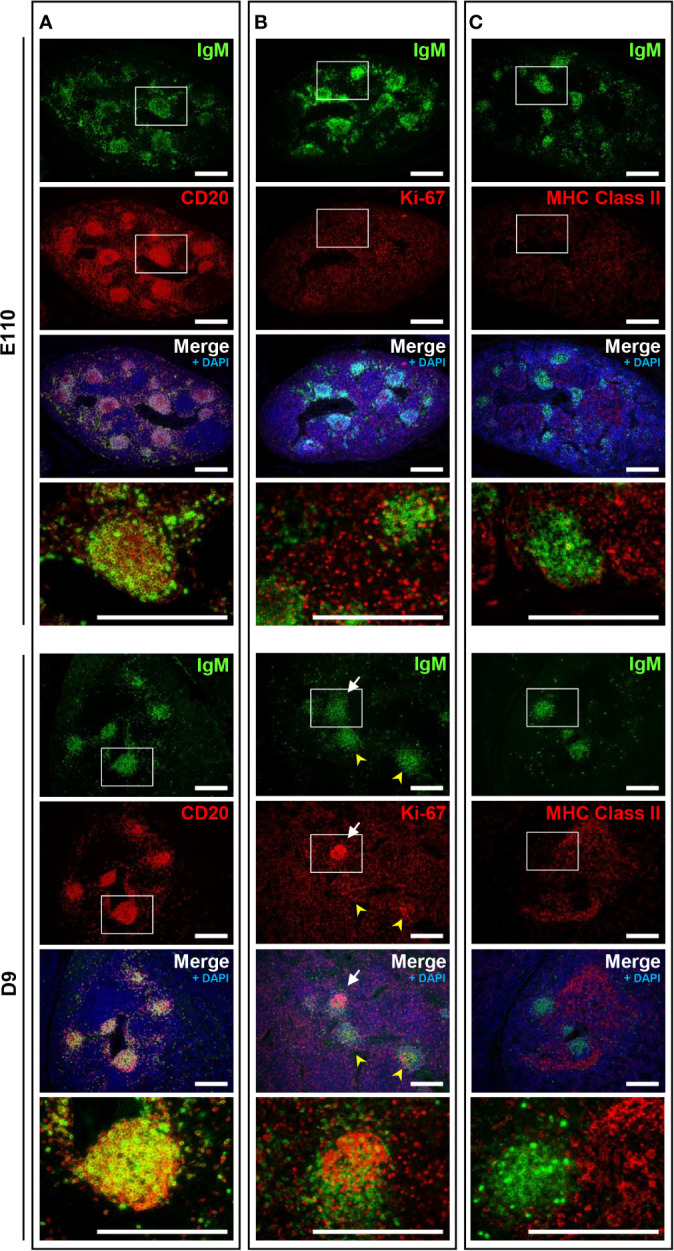
The phenotypic features of B cells in MLNs were not observed on D9 except for the proliferation of B cells in some cortical nodules. **(A)** CD20^+^IgM^+^ cells were found in the cortical nodules at both E110 and D9. **(B)** Some IgM^+^ B cells expressed Ki-67 in a few cortical nodules at D9, but not E110. **(C)** MHC class II^+^ cells were abundant in the cortex surrounding the cortical nodules at E110 and D9. Tissue sections were stained by immunohistochemistry using anti-IgM and either anti-CD20 **(A)**, anti-Ki-67 **(B)**, or anti-MHC class II **(C)**. Scale bars = 250 μm. White arrows: cortical nodules including proliferating IgM^+^ B cells. Yellow arrowheads: cortical nodules without proliferating IgM^+^ B cells.

Because mature B cells in the secondary lymphoid tissues proliferate in response to stimulation by specific antigens via B cell receptors (surface IgM) in the presence of T cells, the immune status of B cells in MLNs was investigated and compared with that of IPPs. Localization of IgM and MHC class II was unchanged postnatally. Specifically, heterogeneously expressed IgM^+^ cells were found mostly in the cortical nodules, and MHC class II^+^ cells were present in the cortex surrounding the cortical nodules at both E110 and D9 (**Figures 6A, B**). A large number of proliferating Ki-67^+^ cells were observed in MLNs at E110; however, they were scattered and not localized to cortical nodules where CD20^+^IgM^+^ B cells were present (**Figure 6C**). By contrast, an accumulation of proliferating Ki-67^+^ cells was seen in some, but not all cortical nodules at D9, which indicated that in several cortical nodules, CD20^+^ B cells expressing different levels of IgM initiated proliferation following antigenic stimulation after birth. It should be emphasized that the simultaneous expansion of B cells in the follicular region of IPPs was not observed in MLNs. These results indicated that MLNs developed as a secondary lymphoid tissue involved in intestinal immune surveillance, but the immune status of MLNs was different from that of IPPs, which directly interact with foreign antigens in the gastrointestinal tract.

### Follicular Regions of IPPs Further Developed Postnatally Through B Cell Expansion and Some Cell Clearance by Apoptosis

**Figure 7:**
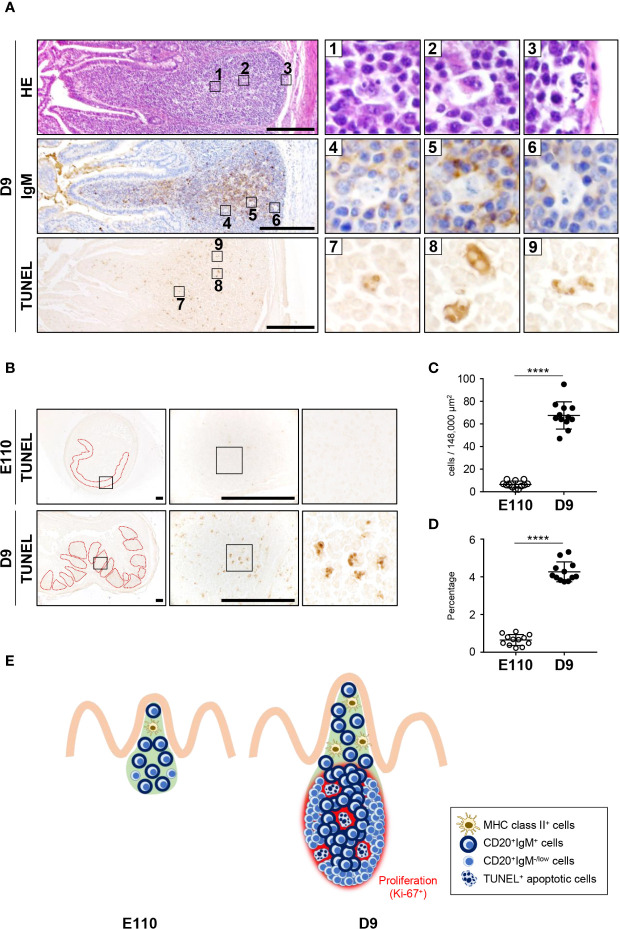
Apoptosis was induced postnatally in the follicular regions of IPPs. **(A)** H&E staining revealed that some cells in the follicular regions of IPPs had abnormal nuclei, lacked expression of IgM and were TUNEL positive on D9. **(B)** Apoptotic cells in IPPs were extremely rare at E110. **(C)** Quantitative analysis indicated a significant difference in the number of TUNEL^+^ apoptotic cells in IPPs between E110 and D9. The number of apoptotic cells was counted in 12 defined areas from seven follicles of three distinct pigs. **(D)** The frequency of apoptotic cells among total cells was calculated. **(E)** Structural and phenotypic differences in IPPs illustrate the postnatal formation of distinct regions containing particular cell populations and apoptotic cells. Tissue sections were stained by H&E and immunohistochemistry using anti-IgM **(A)**, or used for TUNEL assay **(A, B)**. Scale bars = 250 μm. ****p < 0.0001.

To understand the unique characteristics of porcine IPPs, especially after birth, H&E-stained tissues were carefully examined using a high-magnification objective. Importantly, the nuclei of some cells were observed to be lobulated in the follicular regions of IPPs (**Figure 7A**). Additionally, expression of IgM was not detected in cells with lobulated nuclei (**Figure 7A**). Furthermore, subsequent analysis demonstrated that these cells with lobulated nuclei were TUNEL positive (**Figure 7A**), indicating that they were not expressing IgM and were dying by apoptosis in the follicular regions of IPPs. Notably, the number and the frequency of apoptotic cells in IPPs increased significantly after birth, as TUNEL-positive cells were very rare in IPPs at E110, but were readily detected at D9 (**Figures 7B–D**). Taken together, our results indicated that the follicular regions of IPPs developed further postnatally due to the local expansion of B cells and the clearance of some undesired cells, such as cells that do not normally express IgM, by apoptosis (**Figure 7E**).

## Discussion

This study advances knowledge regarding the development of porcine IPP and clarifies the immunological significance of organogenesis involving three sequential steps during both fetal and postnatal stages of development. Over the duration of fetal development in pigs, there was no infiltration of B and T cells in the IPP anlagen during the first two-thirds of pregnancy. However, during fetal development in the last one-third of pregnancy, the expression of CXCL13 and CCL19, which are essential for the recruitment of B and T cells in lymphoid tissues, increased significantly in the ileum, resulting in the migration of both cell lineages into the IPP anlagen (Step 1). Follicular and interfollicular regions of IPPs developed thereafter in fetuses as there was an assembly of B and T cells in distinct regions of IPPs (Step 2). The follicular region of IPPs developed further postnatally through the proliferation of B cells and the induction of apoptosis to exclude some undesired cells, such as cells that do not normally express IgM, in the immune system (Step 3). Considering 114 days of pregnancy in pigs, the timing of initiation of B and T cell migration into the IPP anlagen in step 1 is consistent with the well-known process of PP development in mice, in which organogenesis is initiated in the last one-third of pregnancy (1). Notably, lymphoid tissue inducer (LTi) cells, a subset of type-3 innate lymphoid cells, trigger PP organogenesis in mice and humans (29). LTi cells stimulate residential lymphoid tissue organizer (LTo) cells present in the PP anlagen through the lymphotoxin (LT) α1β2- and LTβ receptor-mediated signaling cascade. This results in the production of several chemokines (i.e., CXCL13, CCL19 and CCL21) and adhesion molecules (i.e., VCAM-1 and ICAM-1) by LTo cells that facilitate the recruitment and retention of migrating B and T cells (30–34). To our knowledge, there are no known reports of studies to characterize porcine LTi and LTo cells or their functions. However, our findings showed that the organogenesis of embryonic IPPs in pigs was concomitant with the increased transcriptional patterns of CXCL13 and CCL19. Therefore, our results indicated that LTi cell-mediated development occurring during the late fetal stages of development may be common in pigs and other species, especially in the early stages of PP organogenesis.

Organogenesis of porcine IPPs and MLNs, both of which comprised gut-associated lymphoid tissues, was compared histologically in this study. Significant differences in developmental processes were found between the two distinct, but related tissues. Few B and T cells were detected in IPPs, whereas those cells were abundant in MLNs at E76. This clearly indicates that lymphopoiesis in the fetal liver is initiated before E76, and that differentiated lymphocytes migrate toward chemokines when secreted from the appropriate anlagen of lymphoid tissues during organogenesis. Given that the initial period of lymphoid organogenesis varies among organs in mice, our finding that MLNs develop earlier than IPPs in pigs is consistent with results of studies using several mouse models (35). Notably, there were no dramatic changes in the numbers of B and T cells in defined regions of MLNs between E76 and D9, although MLNs developed rapidly as body weight increased. By contrast, both the number of B cells in the defined regions of IPPs and the size of the IPPs increased dramatically, indicating that the density of B cells in IPPs increased greatly during organogenesis in IPPs, but not in MLNs. Although the immunological significance of the accelerated development of lymphoid follicles containing a large number of B cells in porcine IPPs needs to be further clarified, our results suggest that B cells in porcine IPPs are actively involved in inducing early stages of the immune response to adapt to the rapidly changing intestinal environment immediately after birth.

Stages of differentiation of B cells vary, and antibody repertoire diversification increases postnatally in porcine IPPs (36). A significant finding of this study regarding PP organogenesis was that porcine IPPs were visibly structured after birth as they form distinct zones in which populations of specific cells reside. Specifically, central, marginal, and subepithelial zones formed in the follicular regions of IPPs at D9. CD20^+^IgM^+^Ki-67^+^ B cells, CD20^+^IgM^−/low^Ki-67^+^ B cells, and antigen-presenting cells that highly expressed MHC class II were abundant in the three distinct zones. By contrast, most CD20^+^ B cells in the follicular regions uniformly expressed IgM, but not Ki-67 at E110, and MHC class II^+^ cells were distributed throughout IPPs without forming obvious subepithelial regions. Generally, the expression of Ki-67 in secondary lymphoid tissues is detected in germinal centers wherein B cells proliferate in the presence of T cell-dependent antigen (37). However, there was extensive expression of Ki-67 in the follicular regions of porcine IPPs at D9, but not at E110 regardless of the absence of germinal center formation (38). Given that multiple toll-like receptors that recognize pathogen-associated molecular patterns are expressed in porcine IPPs, microorganism-derived external stimuli rather than T cell-dependent antigens may accelerate the local expansion of B cells by naturally occurring proliferation. Furthermore, the expression of IgM in CD20^+^ B cells in the marginal zone was extremely low or nearly undetectable although B cells uniformly express IgM in PPs in most other species (39). This difference may be a unique feature of IPPs of pigs, which may require diversification of the antibody repertoire because of the relatively limited number of variable immunoglobulin genes that are rearranged in bone marrow.

Results of the present study indicate that the current definitions of primary and secondary lymphoid tissues are not suitable for IPPs in pigs. M cells are present in the FAE of porcine IPPs (40). Additionally, this study confirmed that MHC class II-expressing cells, presumably dendritic cells (41), are highly abundant in IPPs and form the subepithelial dome region under the FAE. Therefore, luminal antigens may be sampled in porcine IPPs for delivery to dendritic cells to initiate antigen-specific immune responses, which is the primary function of secondary lymphoid tissue. We observed the presence of TUNEL^+^ apoptotic cells and proliferating B cells in the follicles of porcine IPPs, which is not consistent with the definition of secondary lymphoid tissue. In sheep and cattle, IPPs function as a primary lymphoid tissue and apoptotic cells are also found in the follicular regions because of the elimination of B cells that react to self-antigens. Notably, in the follicular regions of IPPs, proliferating Ki-67^+^ cells were apparent before birth in sheep and cattle (42, 43), whereas they were rare before birth in pigs. The difference between pigs and sheep or cattle may be because B cells are prenatally programmed to proliferate in the early stages of differentiation in ovine and bovine IPPs, which are primary lymphoid tissues in those species. Further studies are warranted to precisely define the role of IPPs in pigs. However, our results indicate that porcine IPPs complement a function of primary lymphoid tissue to facilitate the maturation of B cells by creating a niche wherein B cells gain diversity in the presence of external stimuli.

To conclude, results of this study have elucidated developmental processes and identified unique features of porcine IPPs. These new fundamental findings support concepts that will be useful for developing a novel strategy to enhance prenatal and postnatal immune activities that coordinate rapid and dramatic changes in the intestinal microbial environment of newborn piglets.

## Data Availability Statement

The original contributions presented in the study are included in the article/supplementary materials. Further inquiries can be directed to the corresponding author.

## Ethics Statement

The animal study was reviewed and approved by The institutional animal care and use committee of the Institute of Agrobiological Sciences, National Agriculture and Food Research Organization (NARO).

## Author Contributions

MF and TN conceived and designed the study, conducted the experiments, analyzed the data, and wrote the manuscript. SS, DF, and AO maintained pigs, harvested tissues, and conducted the experiments. SI, KN, and KU conducted most experiments and analyzed the data. GW, FB, and KO helped to prepare the manuscript. KW and HA supported experiments and analyses. All authors contributed to the article and approved the submitted version.

## Funding

This study was mostly supported by the Rare/Intractable Disease Project of Japan from The Japan Agency for Medical Research and Development (AMED). This study was also supported in part by the Grants-in-Aid for Scientific Research (A) (18H03969), for the Japan Society for the Promotion of Science (JSPS) fellow (19J11689), and the Core-to-Core Program (Advanced Research Networks) from JSPS, the Program for Interdisciplinary Research from Frontier Research Institute for Interdisciplinary Sciences at Tohoku University, and the Grant for Joint Research Project of Institute of Development, Aging and Cancer, Tohoku University.

## Conflict of Interest

The authors declare that the research was conducted in the absence of any commercial or financial relationships that could be construed as a potential conflict of interest.
